# CD47 as a promising therapeutic target in oncology

**DOI:** 10.3389/fimmu.2022.757480

**Published:** 2022-08-22

**Authors:** Hai Zhao, Shuangshuang Song, Junwei Ma, Zhiyong Yan, Hongwei Xie, Ying Feng, Shusheng Che

**Affiliations:** ^1^ Department of Neurosurgery, the Affiliated Hospital of Qingdao University, Qingdao, China; ^2^ Department of Nuclear Medicine, the Affiliated Hospital of Qingdao University, Qingdao, China; ^3^ Department of Emergency, the Affiliated Hospital of Qingdao University, Qingdao, China

**Keywords:** CD47, SIRPα, immune modulation, immunotherapies, cancer immunotherapy, atherosclerosis, neurological disorders

## Abstract

CD47 is ubiquitously expressed on the surface of cells and plays a critical role in self-recognition. By interacting with SIRPα, TSP-1 and integrins, CD47 modulates cellular phagocytosis by macrophages, determines life span of individual erythrocytes, regulates activation of immune cells, and manipulates synaptic pruning during neuronal development. As such, CD47 has recently be regarded as one of novel innate checkpoint receptor targets for cancer immunotherapy. In this review, we will discuss increasing awareness about the diverse functions of CD47 and its role in immune system homeostasis. Then, we will discuss its potential therapeutic roles against cancer and outlines, the possible future research directions of CD47- based therapeutics against cancer.

## Introduction

**Figure 1:**
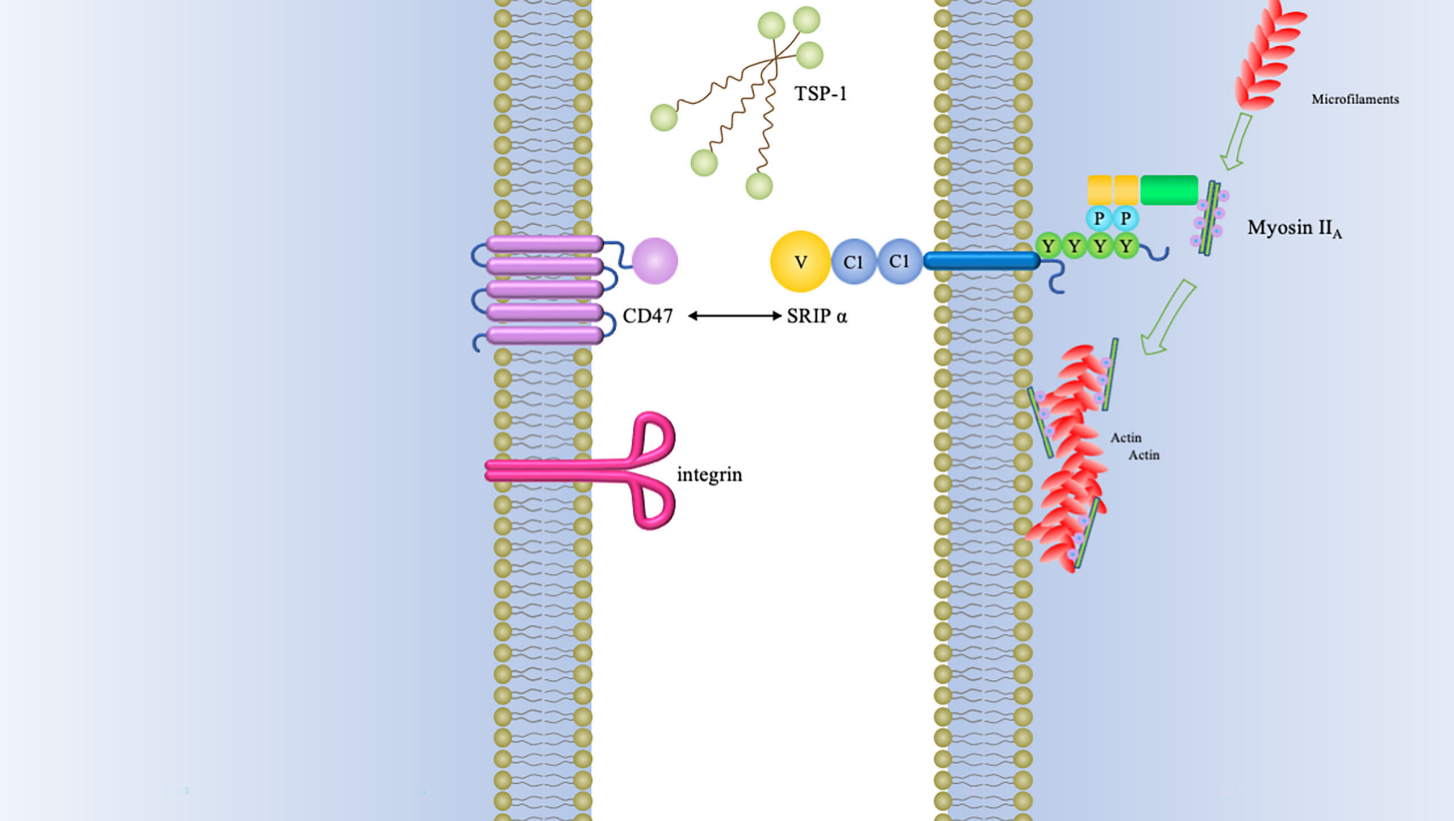
CD47 binding partners and downstream signaling mediators of CD47-SIRPα interaction.The type of signals triggered by CD47 relies on many factors, of which the foremost is the type of ligand that it binds. Three major groups of ligands are identified to exhibit capacity of binding to CD47, namely, SIRPα, TSP-1, and integrins. The interaction between CD47 and SIRPα causes cytoplasmic ITIM phosphorylation and subsequently recruits SHP-1/2. Both of SHP-1 and SHP-2 can inhibit accumulation of myosin-IIA at the phagocytic synapse, which finally inhibit the process of phagocytosis.

CD47 is a membrane receptor glycoprotein composed of a heavily glycosylated N-terminal IgV domain and five transmembrane helices domain with a short cytoplasmic tail (1–3). It was originally identified as a tumor antigen on human ovarian cancer specimens (4). This transmembrane protein is well recognized for its role in “don’t eat me” anti-phagocytic signals by binding to SIRPα (signal regulatory protein alpha) (5). Additionally, TSP-1 (thrombospondin-1) and integrin α2β1 and αvβ3 have also been shown to be CD47 ligands (1, 6) (**Figure 1**).

CD47 is involved in multiple fundamental cellular functions, including cell migration, apoptosis, and axon development (7–9). It is widely expressed on the surfaces of normal cells, especially hematopoietic cells and is best known for its interaction with SIRPα (10). SIRPα is a myeloid-specific immune checkpoint and has been classically described as counterbalanced by a variety of activating membrane receptors (11).The interaction between CD47 and SIRPα emerges as a key regulatory component of innate immune checkpoint by regulating signal transduction and cell clearance. In addition, CD47 is recognized as pivotal for erythrocyte homeostasis, during which both SIRPα and TSP ligands have been shown to be involved (12).

In this article, we will discuss basic functions of CD47 and its associated ligands, review the current knowledge about the involvement of CD47 in host cell phagocytosis, and survey current strategies for developing CD47-based immunotherapy for tumors. In addition, side effects and future research directions for CD47-based immunotherapy will be evaluated.

## Ligands of CD47

The most investigated binding partner of CD47 is SIRPα, which is highly expressed in neurons and a subset of myeloid hematopoietic cells such as dendritic cells (DCs) and macrophages (13). SIRPα is a transmembrane protein comprised of cytoplasmic region with four immunoreceptor tyrosine-based inhibition motifs (ITIMs) and extracellular region with three immunoglobulin (Ig)-like domains (14). The NH_2_-terminal V-like domain of SIRPα is responsible for the binding the extracellular Ig-domain of CD47, whereas ITIMs provide the binding sites for the src homology-2 (SH2)-domain-containing protein tyrosine phosphatases SHP-1 and SHP-2 (15). Recruitment of SHP-1 and SHP-2 phosphatases prevents myosin-IIA accumulation at the phagocytic synapse (16).

By combining different ligands, different signaling pathways can be triggered: When CD47 interacts with SIRPα on the surface of phagocytes, it promotes phosphorylation of the intracellular ITIMs and activates the inhibitory phosphatases SHP-1 and SHP-2, which will inhibit the activation of immune cells by dephosphorylation of proteins containing immunoreceptor tyrosine-based activation motifs (10) (**Figure 1**). Meanwhile, the dephosphorylation cascade initiated by CD47 inside phagocytes lead to deactivation of myosin-II and thereby preventing contractile engulfment (16). Molecular modeling revealed that a VMM motif in C-terminus of TSP-1 is an optimal locus to bind to CD47 (17). TSP-1-CD47 interaction regulates multiple biological functions such as cellular migration, angiogenesis, adhesion, cell aging (18). 4N1K, a peptide corresponding to the CD47 binding region of TSP on IL-12 and TNF-α production, was reported to mediate significant biological effects that were CD47‐independent (19–21). PKHB1, the first‐described serum‐stable soluble CD47‐agonist peptide, directly induced T‐leukemic cell death by engaging the CD47 receptor (20).

CD47 was originally also known as integrin-associated protein (IAP) in earlier studies because of its interaction with aIIbβ3 and a2β1 and αvβ3 integrins (22). In this manner, CD47 functions as a key regulator of migration of smooth muscle cell and platelet activation, etc. (6, 23).

**Figure 2:**
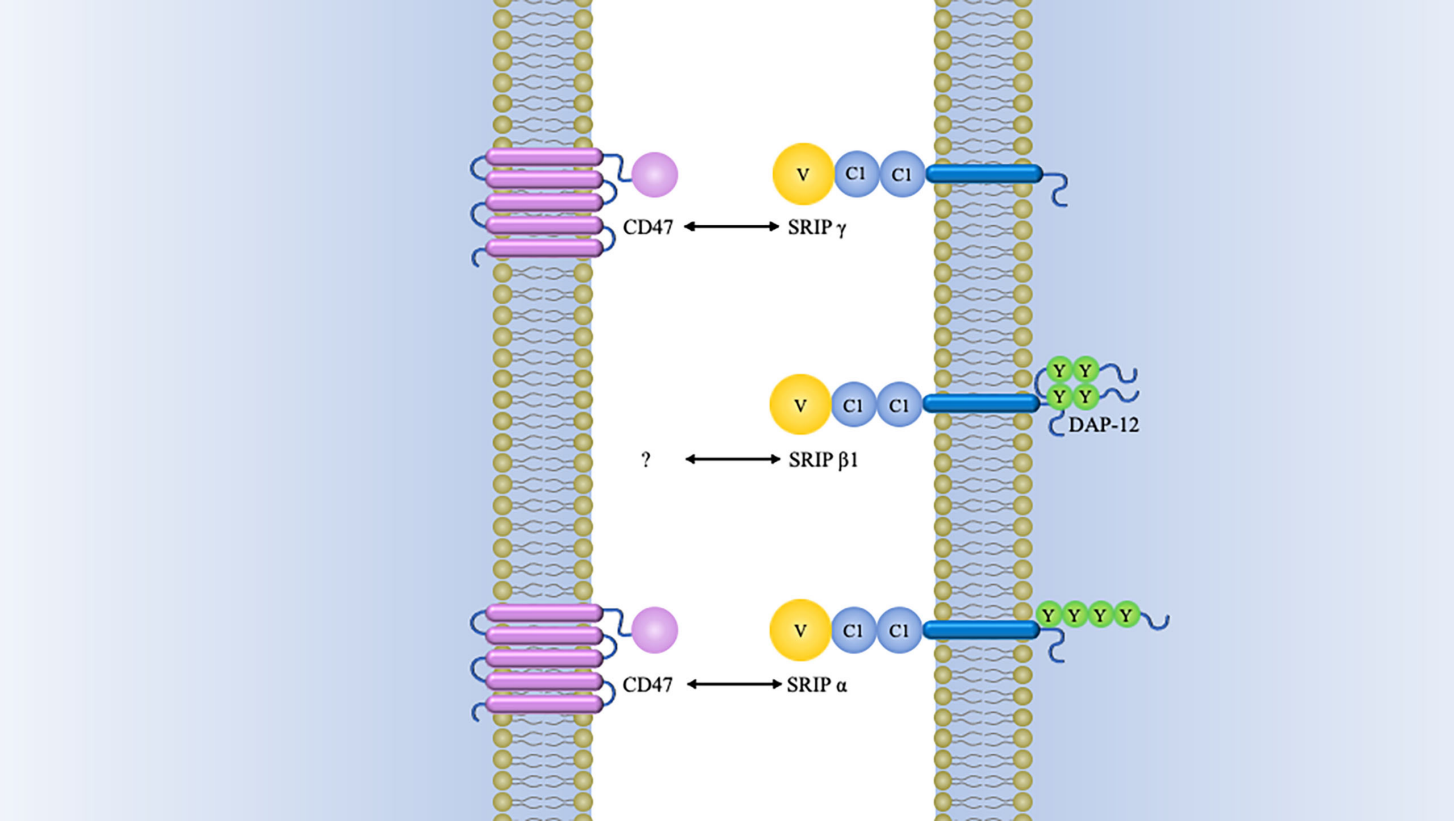
Schematics of structure of SIRP family members. Besides SIRPα, SIRPβ1, and SIRPγ have also been identified in humans. Both of SIRPβ1 and SIRPγ consist of three Ig-like loops in their extracellular domains. SIRPβ1 is characterized by a basic amino acid side chain in its transmembrane domain with a very short cytoplasmic region. This transmembrane region is indispensable for binding of DAP12 (DNAX activation protein 12). It has been established that SIRPβ1 can mobilize the tyrosine kinase Syk, which was followed by MAPK (mitogen-activated protein kinase) activation and microglial phagocytosis enhancement (26). However, it remains unknown what the extracellular ligand for SIRPβ1 and how it might regulate cellular function. There is also a short cytoplasmic region in SIRPγ, but it is quite different from SIRPβ1. The former lacks a charged amino acid residue in its transmembrane region. Nevertheless, SIRPγ can still interact with CD47 by the way of protein-protein binding studies (27). One study has demonstrated that endothelial cell CD47 interacting with SIRPγ plays an important role in T-cell trans-endothelial migration (28). SIRPβ2 is expressed by cells of the monocyte-macrophage lineage and presumably has an association with DAP12 or a similar adaptor (14). SIRPδ has only one domain and has not yet been found any obvious means of membrane attachment (10).

There are other SIRP-family members sharing significantly conserved amino acid sequences within the extracellular domains but different signaling potentials, which are recognized as “paired receptors” (10, 24). In addition to aforementioned SIRPα, the SIRP family have the other members, SIRPβ1, SIRPβ2, SIRPγ, and SIRPδ (13, 25). Among these members, only SIRPγ exhibit a lower affinity with CD47 than that for SIRPα (**Figure 2**).

In addition, several cytoplasmic binding partners of CD47 have been identified, including protein linking IAP and cytoskeleton-1 (PLIC-1), ubiquilin-2 (PLIC-2), vascular endothelial growth factor receptor (VEGFR2), and exportin-1 (18, 29–31). These binding receptors are being exploited in the design of new immune-therapeutic approaches and more robust data are yet to follow on clinical significance of them.

## Host Cell Phagocytosis

**Figure 3:**
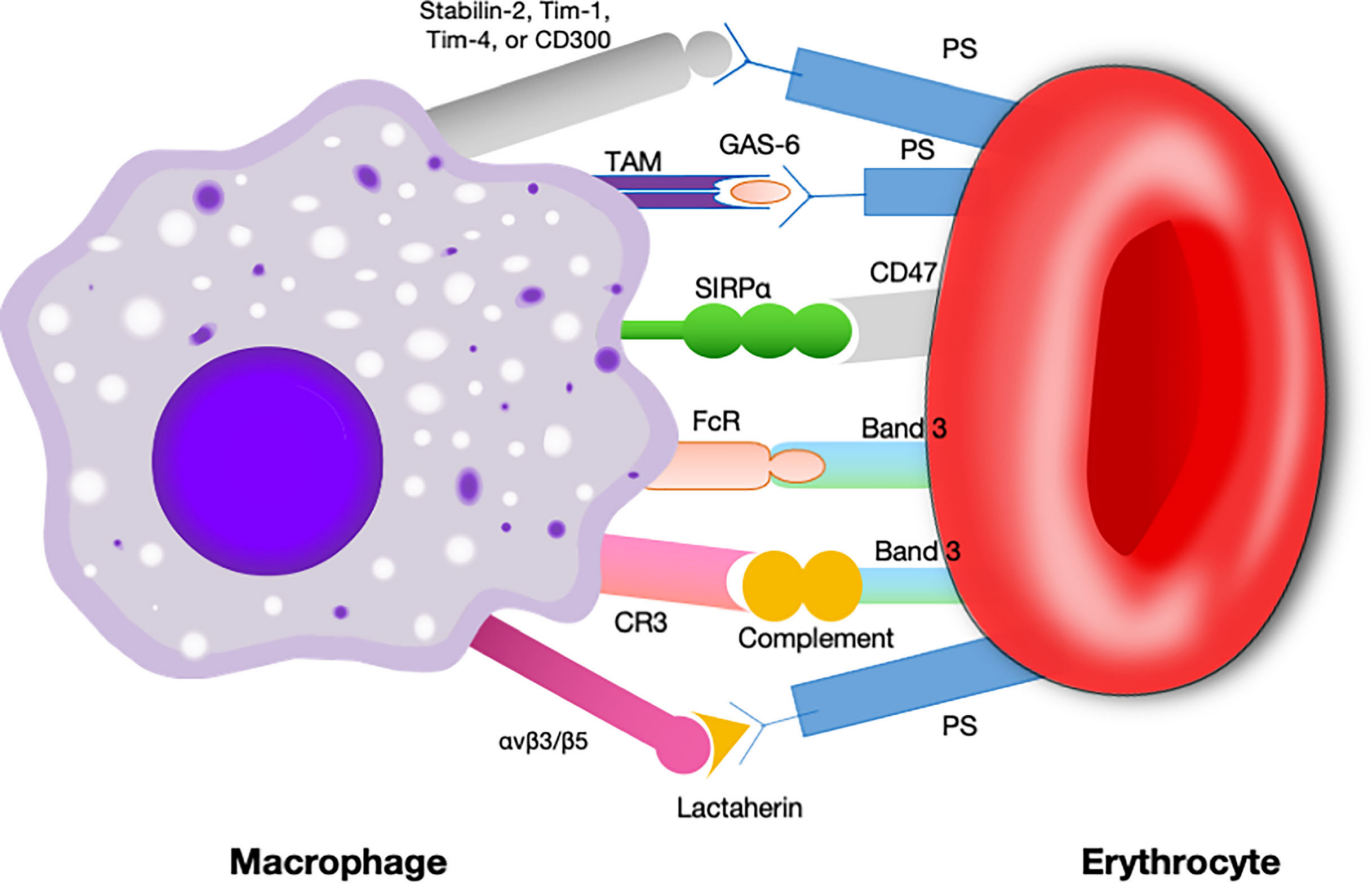
Signals involved in the interaction between macrophages and erythrocytes regulating clearance.Macrophages exhibit some receptors for phosphatidylserine, such as Tim- 1, CD300, Stabilin-2, and Tim-4, which are presumed to deliver a pro-phagocytic signal (34–37). Band 3 is the major target of Nabs. Band 3 clustering and opsonization with Nabs and complements on the erythrocytes facilitates binding to the macrophage *via* Fc receptors and CR-1 and thereby promotes phagocytosis (38, 39). CD47 is a “don’t eat me” signal that plays a crucial role in erythrocytes homeostasis. Binding of CD47 to SIRPα generates an erythrocyte phagocytosis-associated deactivation of myosin IIA, which is a primary contributor to phagocytosis because of its participation in macrophage actinomyosin contraction. Tim, T cell immunoglobulin; Nabs, natural occurring antibodies; CR-1, complement receptor.

In earlier times, CD47-SIRPα interaction was best known for its role as a negative regulator during erythrocytes clearance (32, 33). The original evidence for this came from compelling research in which CD47 knockdown red blood cells, which were quickly cleared by spleen macrophage (33). In contrast, normal erythrocytes can survive for more than 40 days in mice by the reason of CD47 expression (33). Subsequently, it was found that aging erythrocytes have some conformational changes (possibly induced by oxidative modification) in CD47, which change from a “don’t-eat me” configuration into an “eat-me” signal (12). By this token, CD47 not only serves as a pro-phagocytic signal but also behaves as a molecular switch regulating erythrocyte homeostasis (**Figure 3**). This process is thought to be highly associated with Gaucher disease, which is a genetic disorders with glucocerebroside accumulations in cells (40). Comparative analyses of membranous CD47 expression showed that erythrocytes from untreated Gaucher patients have week CD47 expression but can be overturned upon enzyme-replacement remedy (41).

*Kusakari* and colleagues’ study revealed that CD47-SIRPα interaction lead to the internalization of partial membrane by contiguous cells (42). Then it is proved that CD47 did not inhibit, but rather facilitated both phosphatidylserine-independent and phosphatidylserine-dependent uptake of apoptotic cells in the murine system (43). These results confirm prior evidence that CD47 is responsible for removal of some cells rather than just one negative regulator of phagocytosis.

## CD47 and immune cells

### CD47 and effector T cells

CD47 can be found expressed on virtually all immune cells, but the level of expression varies markedly in severity depending on cell types and pathologic conditions (2). For T cells, CD47 signaling is associated with a wide array of cellular processes, from activation to induction of deaths (44). For example, intracellular signaling induced by CD4 stimulates their proliferation, thereby improving the immunological response (45–47). Additionally, CD47 is preferentially expressed on long-lived memory T cell progenitors, which may increase their survival time by preventing clearance by macrophages (48).

CD4^+^ T cells differentiation can be regulated not only by CD47 on themselves but also on antigen presenting cells (APCs). Avice et al. demonstrated that CD47 ligation can selectively inhibit the development of naive T cells into Th1 effectors, which are characterized by production of high levels of IFN-γ, lymphotoxin-a (LT-α), and TNF-α (49, 50). Specifically, inhibiting the expression of IL-12R *via* T cells, impairing the responsiveness of T cells to IL-12, and decreasing IFN-γ production by dendritic cells are among different options how CD47 influence Th1 response (49, 51). Moreover, application of F(ab’)_2_ fragments from CD47-binding peptides or an anti-CD47 mAb has been shown to elicit similar effects (49, 52). The research from *Cham* recently indicated that CD47 blockade enhances T cell responses and speeds lymphocytic choriomeningitis virus clearance (53). These evidences suggest that targeting CD47 holds much promise to treat a wide range of immunity function disturbance with undesirable immune responses.

Liu et al. reveal data suggesting that T cells are required for tumor regression and mediate most of the anti-tumor effects of the CD47 blockade (54). Using syngeneic mouse models of cancer, rather than transplanted xenografts, they first demonstrated that the therapeutic effect mediated by the CD47 blockade is specifically CD8+ cytotoxic T cell–dependent. Phagocytosis of tumor cells by DCs and cross-present antigens to activate downstream CD8+ T initiate adaptive immunity, thereby effectively bridge innate immunity and adaptive immunity. Mechanistically, these anti-tumor effects rely on the cytosolic DNA sensor STING expressed by CD11c+ DCs instead of signaling through MyD88, for example by Toll-like receptors.

### CD47 and Treg Cells

CD47 promotes the differentiation of Treg cells and regulates homeostasis of activated CD103^+^ Treg cells (18, 55); however, a deficiency of CD47 does not alter the inhibitory function of Treg cells (56). Interestingly, naive T cells exhibit an elevated expression of FoxP3 after treatment with anti-CD47/anti-TSP-1, which is akin to the effects of TGF-β and IL-10 (55). CD47 is found highly expressed in the Treg cells in atopic dermatitis patients, suggesting it may contribute to the increased population of Treg cells in this type of eczema (57). In addition, Tregs can protect dopaminergic neurons against MPP+ neurotoxicity underlying CD47-SIRPα interaction (58). This finding provides a novel perspective into how to effect neurodegenerative disease progression by sustaining neuroprotective immunity.

### CD47 and antigen presenting cells

Macrophages are involved in recruitment of immune cells to eliminate foreign materials, aid in tissue repair, and eventually return the tissue to homeostasis (59, 60). CD47 is the dominant macrophage checkpoint, which acts as a “don’t eat me signal” (33). Through its interaction with SIRPα, aged erythrocytes and other ineffective normal cells are quickly cleared by macrophage in the spleen along with accelerated phagocytosis. Further evidence suggest that CD47-SIRPα axis contributes to macrophage activity regulation and alters the polarization state of macrophages (61).

DCs are professional antigen presenting cells with the unique ability to induce naïve T cells activation. They also represent an abundant and stable source of TSP, which can function as an autocrine factor suppressing IL-12 and IFN-γ production by means of interaction with CD47 (62, 63). Meanwhile, the contact between CD47 on T cells and SIRPα on DCs may also participate in the maintenance of immune hemostasis (52). Compared with macrophages, DCs appeared to be the major APCs that cross-prime cytotoxic T cells following CD47 blockade since the therapeutic effect was severely impaired after DC depletion rather than macrophages (54). The authors attributed this different efficiency to higher expression of *Ifna* mRNA on DCs (54).

### CD47 and nature killer cells

CD47 plays negative roles in NK cells activation and proliferations upon binding its ligand TSP-1, while lack of CD47 increase NK cells activation and cytotoxicity (64, 65). As a SIRPα counter-receptor, CD47 is involved in NK precursors engraftment in humanized mice (66, 67). In tumor microenvironment, CD47 impairs the recruitment of NK cells, whereas treatment with anti-CD47 antibody increases NK cells killing against tumor cells by enhancing expression of granzyme B and IFN-γ (65). Though future studies are still needed to better understand the intricate mechanisms recruiting NK cells, targeting CD47 has a therapeutic potential as a NK cell checkpoint in tumor micro-environment (68).

### CD47 and other cell types

Emerging evidences indicate that CD47 plays an important role in trans-endothelial migration of neutrophil and other leukocytes (69, 70). Diverse anti-CD47 mAbs have been illustrated to suppress neutrophil movements across cell mono-layers *in vitro* (7, 71, 72). Additionally, neutrophil mobilization was shown to be retarded *in vivo* in CD47-deficient mice by means of intraperitoneal inoculation of *Escherichia coli* (70). Also, recent studies demonstrated that CD47 molecule expressed on myeloid DCs is a critical factor in controlling efficiently traffic across lymphatic and endothelial vessels, seeding in secondary lymphoid organs and participating in T-cell priming (54, 73).

## Targeting CD47 for cancer immunotherapy

In the current era in oncology, checkpoints immunotherapy of hematopoietic and solid malignancies are becoming a promising mode of treatment for cancer patients (74, 75). But the fact that not all cancer patients benefit from adaptive checkpoints immunotherapy catalyzed enormous interests in the targeting of novel immune checkpoint receptors. Considering innate immune system is the first line of defense directly target cancer cells, harnessing innate immunity represents another potential therapeutic avenue for cancer treatments. Innate checkpoints have recently received some attention regarding their possible roles in tumor-mediated immune escape. A list of innate checkpoints of interest are under investigation, including CD47-SIPα axis, TAM family (Tyro3, Axl, and MerTK), Siglec-9, CD24-Siglec-10 axis (76–79).

### Mechanisms of action of CD47 targeted therapy

**Figure 4:**
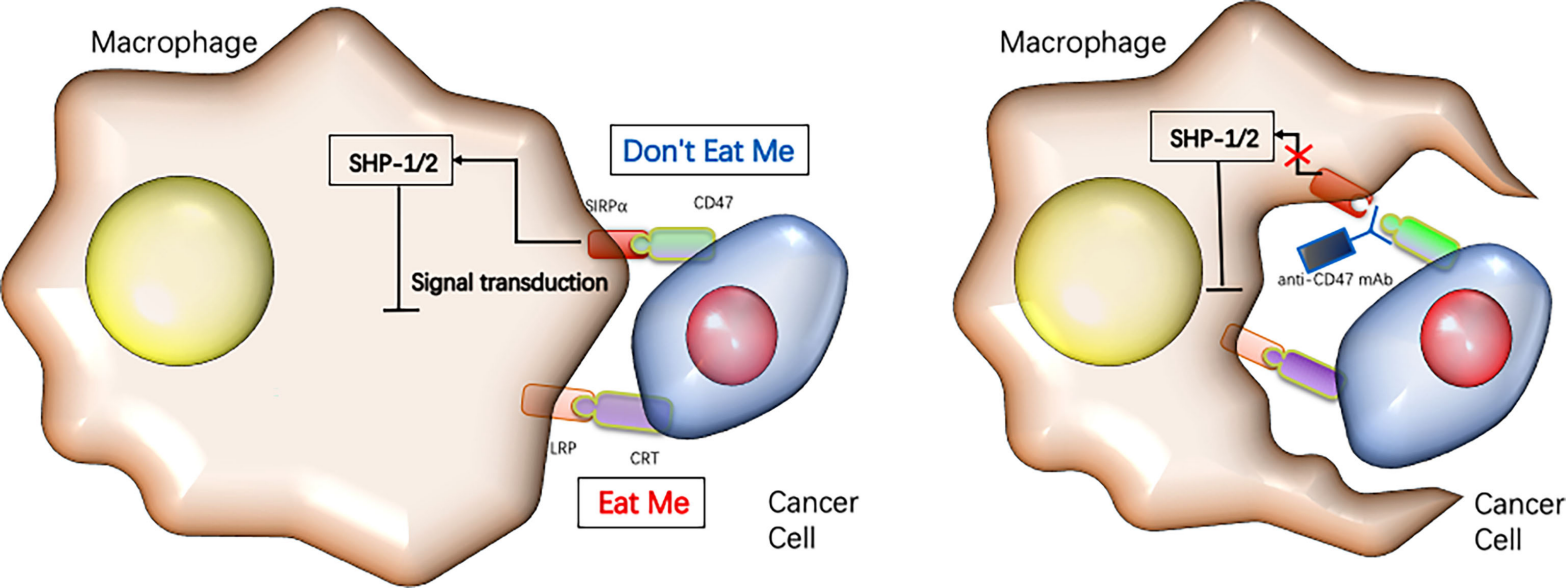
Targeting CD47 immune checkpoint for cancer immunotherapy.Cancer cells rely on the expression of “don’t eat me” signals such as CD47 to inhibit their phagocytotic clearance by macrophages, while blocking CD47 reduces tumor growth by enabling macrophages to phagocytose the cancer cells [reviewed in refs (22)].

**Figure 5:**
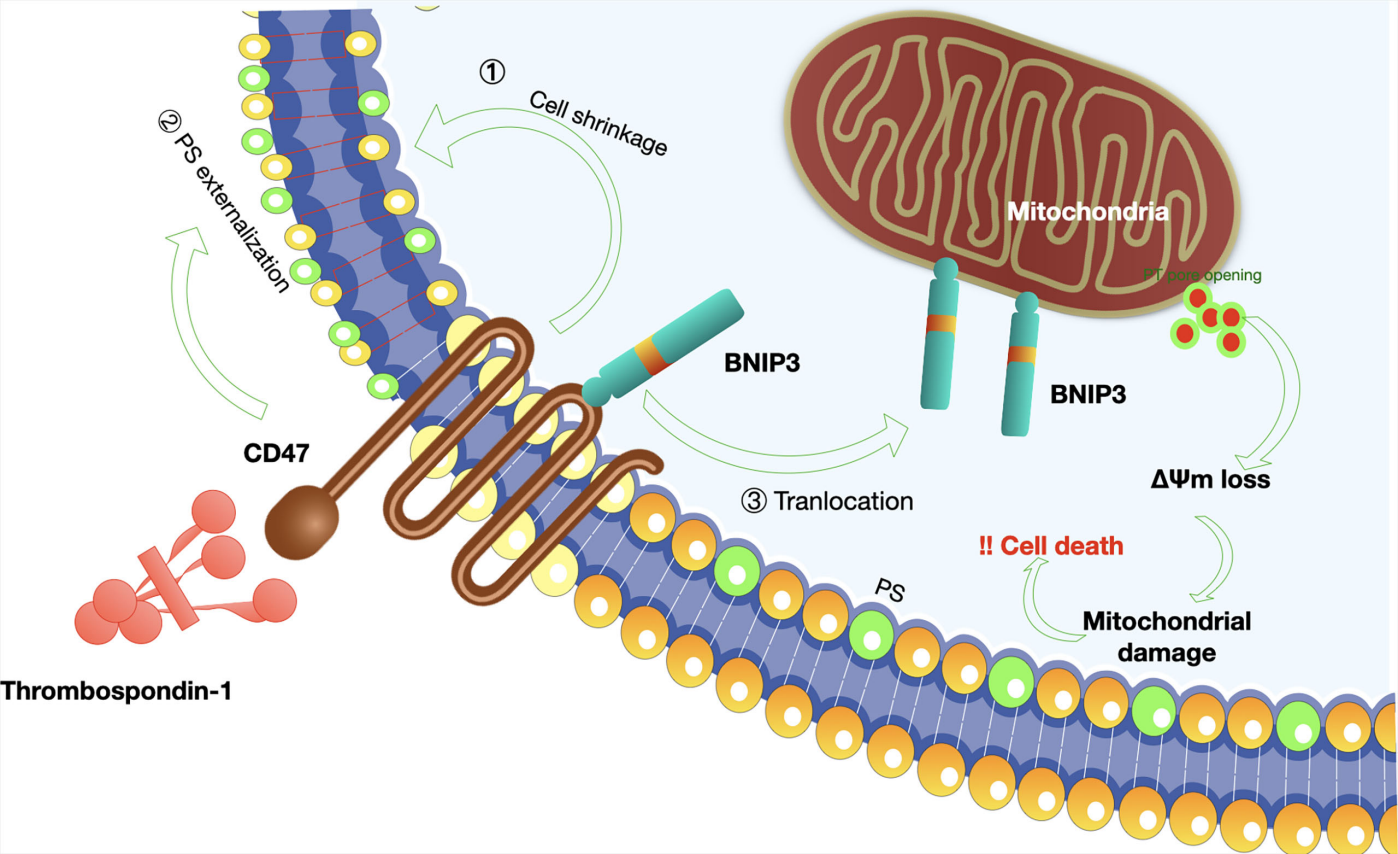
Mechanisms of CD47-induced caspase-independent cell death.CD47 induces a caspase-independent cell death, characterized by intact or slightly modified nuclei, reduced cell viability (80). The process of cell apoptosis includes PS (phosphatidylserine) exposure, disruption of mitochondrial function, and cytoskeleton rearrangement (1): K+ is a critical component of volume regulatory response, and leakage of K+ is a simple way to accommodate a rapid decrease in cell volume; CD47-induced cell death was dependent on K+ efflux (81); (2) Mechanisms involved in PS exposure remain poorly understood. Veronique M et al. proposed that CD47 ligation initially induce triggering of actin polymerization, perhaps *via* Cdc42/WASP pathway. This event then leads to mitochondrial changes including matrix swelling and ΔΨm (mitochondrial transmembrane potential) loss, followed by PS exposure or bypass of mitochondria and direct induction of PS externalization (81); (3) In addition, Bcl-2 homology 3 (BH3)-only protein 19 kDa interacting protein-3 (BNIP3) is indispensable for the pro-apoptotic effect of CD47 (82). A simple model-based on the translocation of BNIP3 from the inner surface of the cell membrane to the mitochondria is proposed since BNIP3 can only exert its pro-apoptotic effect at the mitochondria membrane (83).

The theoretical basis for CD47 functioning as a promising checkpoint in cancer therapy is due to its pivotal role in balancing both inhibitory and stimulating activities of myeloid cells (**Figure 4**). Firstly, CD47 ligation induce apoptosis of tumor cell through a caspase-independent mechanism (**Figure 5**) (84, 85). Secondly, anti-CD47 leads to tumor cell phagocytic uptake by antigen presenting cells and subsequent antigen presentation to T cells (76, 86). Thirdly, anti-CD47 abrogates the TSP-1-mediated inhibitory effect against human NK cells but increase NK cells activation and cytotoxicity (65). CD47 blockades have shown initial success in early- phase clinical trials for many human cancers, either alone or in combination with other agents (**Table 1**) (87, 88). Fourthly, preclinical study by Xiaojuan L et al. demonstrated that therapeutic effect of CD47 blockade depends on STING (stimulator of interferon genes), which induces a type I/II IFN response mediated by dendritic cells and CD8+ T-cells (54). Fifthly, some literatures revealed that CD47/TSP-1 pathway also has pleiotropic effects on immunity system and may present a new target for potential cancer therapeutics (89, 90).

**Table 1:** Registered clinical trials on clinicaltrials.gov focused on CD47-SIRP axis.

Sponsor (Lead Drug)	Clinical Trials.gov Identifier	Intervention	Cancer type	Phase
Gilead Magrolimab	NCT02953509	+ Rituximab	Relapsed/Refractory B-cell Non-Hodgkin Lymphoma	I/II
	NCT02953782	+ Cetuximab	Solid Tumors and advanced colorectal cancer;	I/II
	NCT03248479	Monotherapy or + Azacitidine	Hematological Malignancies	I
	NCT03558139	+ Avelumab	Ovarian Cancer	I
Celgene CC-90002	NCT02641002	Monotherapy	Acute myeloid leukemia; myelodysplastic syndromes	I
	NCT02367196	Monotherapy or + Rituximab	Hematologic Neoplasms	
Surface Oncology SRF231	NCT03512340	Monotherapy	Advanced Solid and Hematologic Cancers	I
ALX Oncology ALX148	NCT03013218	Monotherapy or + Rituximab /Pembrolizumab /Trastuzumab	Advanced Solid Tumors and Lymphoma	I
Trillium TTI-621	NCT02663518	Monotherapy or +Rituximab /Nivolumab	Hematological Malignancies and Selected Solid Tumors	Ia/Ib
	NCT02890368	Monotherapy or + PD1/PD-L1 Inhibitor /pegylated interferon-α2a /T-Vec/radiation	Relapsed and Refractory Solid Tumors and Mycosis Fungoides	I
Trillium TTI-622	NCT03530683	+Rituximab	Relapsed or refractory lymphoma or myeloma	I
Arch Oncology AO-176	NCT03834948	Monotherapy	Multiple solid tumor malignancies	I
Innovent Biologics IBI188	NCT03763149	Monotherapy	Advanced malignant tumors and lymphomas	I
	NCT03717103	Monotherapy or + Rituximab	Advanced Malignancies	I

### Molecular mechanisms underlying anti-CD47 therapy

Whether blocking CD47 alone is sufficient to induce phagocytosis is a matter of dispute (91, 92). Some evidence supports that targeting CD47 would be sufficient for elimination of tumor cells by macrophage (93, 94). It should be noted that most of the antibodies tested before include an Fc region, which recognizes Fc receptor γ-chain (FcRγ) on macrophage, neutrophils, and NK cells (95, 96). On one hand, activating FcRs associated with FcRγ of immunoreceptor tyrosine-based activation motif (ITAM) and, thereby, trigger phagocytosis of macrophage (96). On the other hand, the interaction between Fc region and FcRs simultaneously opsonize the target cells for antibody-dependent cell-mediated cytotoxicity (ADCC) since NK cells are the crucial mediators of this reaction *in vivo* (87, 97). Moreover, FcR-activated NK cells can also secrete IFN-γ, TNF-α, and lead to increased expression of interleukin-21 receptor that suppress tumor growth (98, 99). Therefore, insights raise intriguing questions as to whether anti-tumor effects induced by CD47-antibodies occur through Fc-dependent mechanisms? Which acts as the predominant signal responsible for anti-tumor effects, modulation of phagocytosis or ADCC?

It is of great significance to the development of therapeutic agents because of balance between their direct killing effect against cancer cells and their potentially hazardous side effects on normal CD47-positive cells. If ADCC generally dominates during the stage of immune response, tolerable toxicity can be achieved with an impaired therapeutic efficacy (100).

To minimize the effect of Fc-dependent effector functions, investigators designed a chimeric antibody (Hu5F9-G4) containing the Fc portion that binds to FcRs with a lower affinity compared to other human IgG subclasses (101). ADCC and complement- dependent cytotoxicity (CDC) activities were tested, and Hu5F9-G4 was unable to induce ADCC and CDC at any of the concentration tested (101). But it still triggered potent macrophage-mediated phagocytosis against human hematological cells, suggesting that CD47-SIRPα blockade alone may be sufficient to induce tumor regression (101). However, the application of the agent having lower affinity with FcRs did not completely exclude the direct ADCC since even minimal engagement of FcRs could be enough to activate signaling cascade of phagocytosis.

The best way to completely exclude direct ADCC is to use F(ab’)_2_ fragment antibodies. F(ab’)_2_ fragment is characterized with removal of most of the Fc fragment, while leaving the hinge region intact. Research data showed that anti-CD47 F(ab’)_2_ fragment was able to promote engulfment of Raji and other lymphoma cells *in vitro* (88, 102). Intriguingly, this phenomenon of engulfment activation was not applicable to Jurkat and various colon carcinoma cell lines (91, 102). Studies later confirmed that the first group of tumor cells expressed other phagocytic receptors that can bypass the need for FcR engagement (88).

CD47 antibodies with devoid of Fc portion are theoretically an ideal candidate for the development of therapy approaches to modulate CD47-SIRPα interaction. This can be achieved *via* the form of nanobody or single-chain variable fragment (103). For example, HuNb1-IgG4, an innovative anti-CD47 nanobody, has been developed to reduce adverse effects of blocking CD47-SIRPα interaction (103). It was shown that HuNb1-IgG4 presented potent anti-tumor activities *in vivo*, and more importantly, it exhibited high safety for hematopoietic system in cynomolgus monkeys (103).

As is discussed before, CD47-SIRPα blockade alone, in most cases, is not sufficient for the induction of significant phagocytosis and subsequent antitumor activity. A secondary stimulus, such as other prophagocytic signal (i.e., calreticulin, SLAMF7, and macrophage-1 antigen) or an opsonizing antibody (i.e., rituximab) is indispensable (104). Calreticulin is proposed to be a pro-phagocytic signal recognized by lipoprotein receptor-related protein-1 (LRP-1) on macrophage (105, 106). SLAMF7 (signaling lymphocytic activation molecule family 7, also known as CRACC, CS1, and CD319) remarkably facilitates engulfment of a few hematopoietic tumor cells expressing SLAMF7 such as Raji during CD47-SIRPα blockade (45, 102). This explains why anti-CD47 F(ab’)_2_ fragment was able to promote engulfment of *Raji* (107). In addition, SLAMF7 interact with macrophage-1 antigen (MAC-1) to form a protein complex on the macrophage surface (102). The complex consists of two ITAM-containing receptors, FcRγ and DAP12, which trigger the phagocytic machinery through SRC kinase, spleen tyrosine kinase, and Bruton’s tyrosine kinase (102). All the three prophagocytic receptors can be exploited to enhance anti-tumor response during CD47-SIRPα blockade.

### Small-molecule inhibitors targeting CD47

In some aspects, small-molecule agents are superior to therapeutic antibodies since they can increase active time period and absorbability in the body (108). Research on small-molecule inhibitors targeting CD47 has also been one of the focuses for cancer immunotherapy. These inhibitors can modulate CD47 at the transcriptional, translational, and post-translational modification levels. In a concentration-dependent manner, both NCGC00138783 and pep-20 bind to CD47 and exert their blocking functions (109, 110). Some small-molecule agents can suppress CD47 expression at transcriptional or translational levels, including RRx-001, metformin, 4-methylumbelliferone, JQ1, and gefitinib (111). Excitedly, RRx-001 has showed potent anti-cancer activities *via* different pathways and has been proceeded into Phase III clinical trial (NCT03699956) (112, 113). At the post-translational modification level, QPCTL (glutaminyl-peptide cyclo-transferase-like protein) disrupts CD47 pyroglutamate formation and is regarded a novel target to augment antibody therapy of cancer (114, 115). To sum up, there have been big progress in this endeavor recently and this field deserves increased attention in future.

## Side effects of anti-CD47 treatments and proposed solutions

**Figure 6:**
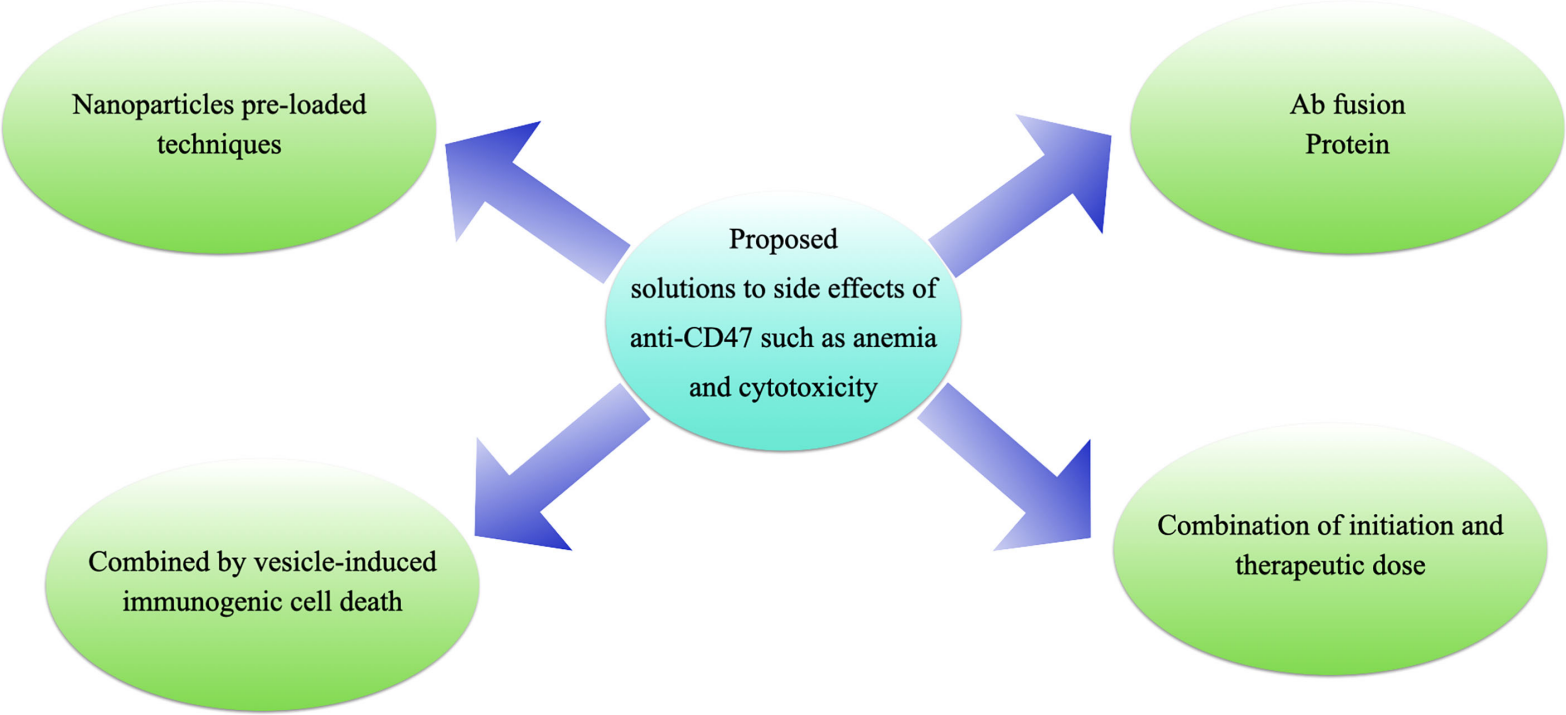
Proposed solutions to the side effects of anti-CD47 treatments. **(A)** antibody fusion proteins, e.g., TTI-621 (116) or ALX-148 (26); **(B)** “initiation doses” followed by “therapeutic doses”, e.g., Hu5F9-G4 (117); **(C)** pro-drug vesicle-induced immunogenic cell death combined with CD47 blockade (54); or **(D)** tumor-targeting nanoparticles loaded with anti-CD47 antibody (27).

Given the ubiquitous expression of CD47 on normal cells, potential side effects such as anemia will need to be considered though several clinical trials are underway and have produced impressive clinical results in recent years (**Figure 6**).

In several preclinical solid tumor models, tumor-specific delivery of CD47 blockade demonstrated superior accumulation and fewer side effects at tumor sites compared with systemic administration (118) (119). Novel drug delivery system has emerged as a critical modulator in the development of anti-CD47 therapeutics, such as nanomedicine and gel matrix (117, 120–122).

Bispecific antibodies are helpful for the purpose to limit bystander effects due to widespread CD47 expression. Heretofore, some bispecific antibodies have been developed with one arm binding and blocking CD47 to prohibit its interaction with SIRPα, and the other arm binding tumor-associated antigens such as CD19, CD20, and PD-L1 (123). In comparison to other CD47 antibodies, bispecific antibody was engineered to have a lower affinity towards CD47, limiting binding activity with normal cells expressing CD47. Here, proof was provided by an anti-CD47/CD19 bispecific antibody that selectively block the CD47-SIRPα on malignant cells expressing CD19 and interact weakly with normal cells expressing CD47 (124). Such a bispecific design could be applied to limit the negatively impacts caused by “antigen sink”.

In addition to drug delivery system and bispecific antibody, there are many other strategies to prevent anemia caused by anti-CD47 therapeutics: gradually increase therapeutic dose (125); differentiate erythrocytes CD47 and tumor cell-expressing CD47 (114, 126–130); sacrifice antibodies mediated ADCP (antibody-dependent cellular phagocytosis) (131–133); pro-body technology (134) (120). Apart from anemia, issues with thrombocytopenia, hyperbilirubinemia, and neutropenia have also limited the use of anti-CD47. For further reading on this field, we recommend to the curious reader one excellent review by Yuchen W et al. (135). The review also high-light recent advances in tumor therapy targeted on the CD47-SIRPα axis and provides ideas for further clinical transformation.

## Future perspective

Although early data with agents targeting either CD47 or SIRPα have highlighted the therapeutic potential by sending a potent “don’t eat me” signal to prevent phagocytosis, no Phase III study convincingly demonstrated the efficacy of these novel therapeutic targets for cancer treatment so far. ENHANCE (NCT04313881), a randomized, double-blind, placebo-controlled multicenter Phase III study from Forty-Seven (Gilead Sciences) aimed to compare the effects of treatment with magrolimab plus azacitidine and placebo plus azacitidine in untreated patients with myelodysplastic syndrome, has recently resumed after FDA lifted the partial clinical hold placed on the item. This is the only Phase III clinical trial related to CD47 blocking therapy. Inhibitors and antibodies against CD47 in immunotherapy have several limitations to emerge, and some features of the therapy must be considered.

The first limitation is to search for safe potent antibodies that are not highly bound to erythrocytes at early step in terms of safety. Earlier detection of the potential toxicity during antibody development can help cut costs and improve success rate. Dual targeting bi-specific antibodies of both TAA (tumor-associated antigens) and CD47 can also be designed to resolve this issue, which can direct CD47 blockade selectively to cancer target cells, thereby improving the safety of CD47 blocking therapy (136) (137).

Secondly, immunotherapy with CD47 blockade has achieved impressive successes in treatment of hematological malignancies. But the clinical efficacy of CD47 blockades in solid tumors has been much less rewarding due to the tumor heterogeneity and intricacies of the tumor microenvironment. Compared with macromolecule agents, small molecule inhibitors are more penetrative to the targets and have broad prospects for development and application in solid tumors (138). For example, a humanized CD47/GPC3 (Glypican-3) BsAb enhances the Fc-mediated effector function and has significantly better efficacy than the combined treatment using CD47 and GPC3 monoclonal antibodies in the hepatocellular carcinoma model of xeno-transplantation (139). Apart from bispecific antibodies, nanobodies have also received widespread attention due to their deeper penetration in solid tumors. HuNb1-IgG4, a new type of CD47 nano-antibody that has a low affinity for human erythrocytes, enhances tumor phagocytosis mediated by macrophages *in vitro*, and shows strong anti-tumor activity in ovarian cancer and lymphoma (103). Thirdly, CD47 can also be used in other therapeutic areas, including atherosclerosis, neurological disorders, and autoimmune diseases. By administration of CD47-blocking antibodies, the atheroprone mice would develop significantly smaller atherosclerotic plaques compared to IgG controls (140). A potential role for CD47 in Alzheimer’s disease has been identified with studies showing CD47 facilitates Aβ oligomers internalization by microglia (141, 142). Even in the infancy of understanding its diverse function and potentiality, CD47 will be further revealed as an important mediator in these therapeutic areas.

## Conclusions

Inspiring progress has been made with respect to the understanding of the role of CD47 in normal cell growth cycle as well as in the molecular pathogenesis of diverse diseases. CD47 serves as a receptor for TSP, integrins, and SIRP family members and has been shown to trigger a wide variety of cellular functions. For example, several lines of evidence suggests CD47 ligation appears to co-stimulate T cell proliferation and induce their arrest (54).

Utilizing anti-CD47 antibodies open up exciting avenues in cancer, but there are still unsolved questions in this field. First, the underlying mechanisms need to be elucidated. What is the importance of FcR engagement during CD47 blockade? How to avoid an uncontrolled immune response since the ubiquitous expression of CD47? The cell specific function of CD47 is not fully understood so far and its downstream signaling is still unclear. Secondly, there exists more than one binding receptor of CD47. It, therefore, would be challenging to decide what are the specific contribution of each partner in this intricate network? Thirdly, the question remains whether NK cells or other immune cells contribute to the effect of CD47 blockade rather than macrophage. Finally, some researchers asserted that anti-CD47 antibody did not induce apparent side effects in animal models, but whether if it is equally safe in human body still needs further elucidation. In conclusion, though there is still long way to go about mechanistic issue of CD47 blockade, immunological and clinical studies are yielding impressive results in this field. An exciting new era of innate checkpoint strategy targeting CD47 is likely to come especially when we fully understand underlying mechanisms.

## Author contributions

All authors listed have made a substantial, direct, and intellectual contribution to the work, and approved it for publication.

## Conflict of interest

The authors declare that the research was conducted in the absence of any commercial or financial relationships that could be construed as a potential conflict of interest.

## Publisher’s note

All claims expressed in this article are solely those of the authors and do not necessarily represent those of their affiliated organizations, or those of the publisher, the editors and the reviewers. Any product that may be evaluated in this article, or claim that may be made by its manufacturer, is not guaranteed or endorsed by the publisher.
